# Supplementary material for: Comparative Analysis of the Immune Response and the Clinical Allergic Reaction to Papain-like Cysteine Proteases from Fig, Kiwifruit, Papaya, Pineapple and Mites in an Italian Population
Source: Foods. 2023 Jul 27;12(15):2852. doi: 10.3390/foods12152852 (PMC10417190; doi:10.3390/foods12152852)
Supplement: Supplementary file 1 [file foods-12-02852-s001.zip › foods-2489397-supplementary.pdf]

foods

MDPI

Comparative Analysis of the Immune Response and the Clinical Allergic Reaction to Papain-like Cysteine Proteases from Fig, Kiwifruit, Papaya, Pineapple and Mites in an Italian Population

Ivana Giangrieco [1], Maria Antonietta Ciardiello [1,*], Maurizio Tamburrini [1], Lisa Tuppo [1], Chiara Rafaiani [2], Adriano Mari [2,3] and Claudia Alessandri [2,3]

1 Institute of Biosciences and BioResources (IBBR), National Research Council of Italy (CNR), 80131 Naples, Italy; ivana.giangrieco@ibbr.cnr.it (I.G.); maurizio.tamburrini@ibbr.cnr.it (M.T.); lisa.tuppo@ibbr.cnr.it (L.T.)
2 Associated Centers for Molecular Allergology (CAAM), 00100 Rome, Italy; pedrigna@gmail.com (C.R.); adriano.mari@caam-allergy.com (A.M.); claudia.alessandri@caam-allergy.com (C.A.)
3 Allergy Data Laboratories (ADL), 04100 Latina, Italy
* Correspondence: mariaantonietta.ciardiello@ibbr.cnr.it

```
CLUSTAL O(1.2.4) multiple sequence alignment


sp|P14518|BROM2_ANACO       ------------------------------------------------------------      0
tr|O23799|O23799_ANACO      MAWKVQVVFLFLFLCVMWASPSAASADEPSDPMMKRFEEWMVEYGRVYKDNDEKMRRFQI      60
tr|O81084|O81084_ANACO      MAWKVQLVFLFLFLCVMWASPSAASADEPSDPMMKRFEEWMVEYGRVYKDNDEKMRRFQI      60
tr|O23800|O23800_ANACO      MASKVQLVFLFLFLCVMWASPSAASRDEPSDPMMKRFEEWMAEYGRVYKDNDEKMRRFQI      60
tr|F1KD58|F1KD58_ANACO      MASKVQLVFLFLFLCVMWASPSAASRDEPSDPMMKRFEEWMAEYGRVYKDNDEKMRRFQI      60
tr|Q7DNA3|Q7DNA3_ANACO      ----------------------------------------MAEYGRVYKDNDEKMRRFQI      20
tr|O24641|O24641_ANACO      MASKVQLVFLFLFLCVMWASPSAASRDEPSDPMMKRFEEWMAEYGRVYKDNDEKMRRFQI      60
sp|O23791|BROM1_ANACO       MASKVQLVFLFLFLCAMWASPSAASRDEPNDPMMKRFEEWMAEYGRVYKDDDEKMRRFQI      60
tr|O23801|O23801_ANACO      ---------------------------EPNDPMMKRFEEWMAEYGRIYKDNDEKMRRFQI      33
tr|F8UN02|F8UN02_ANACO      MASKVQLVFLFLFLCVMWASPSAASRDEPSDPMMKRFEEWMAEYGRVYKDNDEKMRRFQI      60
tr|L7UXZ2|L7UXZ2_ANACO      ------------------------------------------------------------      0
sp|P80884|ANAN_ANACO        MTSKVQLVFLFLFLCVMWASPSAASCDEPSDPMMKQFEEWMAEYGRVYKDNDEKMLRFQI      60
tr|S5VRY7|S5VRY7_ANACO      MASKVQLVFLFLFLCVMWASPSAASRDEPSDPMMKRFEEWMAEYGRVYKDNDEKMRRFQI      60
tr|O81085|O81085_ANACO      MASKVQLVFLFLFLCVMWASPSAASRDEPSDPMMKRFEEWMAEYGRVYKDNDEKMRRFQI      60


sp|P14518|BROM2_ANACO       ------------------------------------------------------------      0
tr|O23799|O23799_ANACO      FKNNVNHIETFNSRNENSYTLGINQFTDMTNNEFIAQYTGGISRPLNIEREPVVSFDDVD      120
tr|O81084|O81084_ANACO      FKNNVNHIETFNSRNKDSYTLGINQFTDMTNNEFVAQYTGGISRPLNIEREPVVSFDDVD      120
tr|O23800|O23800_ANACO      FKNNVNHIETFNNRNGNSYTLGINKFTDMTNNEFVTQYTG-VSLPLNFKREPVVSFDDVN      119
tr|F1KD58|F1KD58_ANACO      FKNNVNHIETFNNRNGNSYTLGINKFTDMTNNEFVAQYTGGISRPLNIEKEPVVSFDDVN      120
tr|Q7DNA3|Q7DNA3_ANACO      FKNNVNHIETFNNRNGNSYTLGINKFTDMTNNEFVAQYTGGISRPLNIEKEPVVSFDDVN      80
tr|O24641|O24641_ANACO      FKNNVNHIETFNNRNGNSYTLGINKFTDMTNNEFVAQYTGGISRPLNIEKEPVVSFDDVN      120
sp|O23791|BROM1_ANACO       FKNNVKHIETFNSRNENSYTLGINQFTDMTKSEFVAQYTG-VSLPLNIEREPVVSFDDVN      119
tr|O23801|O23801_ANACO      FKNNVKHIETFNSRNGNSYTLGINQFTDMTKSEFVAQYTG-VSLPLNIEREPVVSFDDVN      92
```

```
tr|F8UN02|F8UN02_ANACO      FKNNVNHIETFNSHNGNSYTLGINQFTDMTKSEFVAQYTGGISRPLNIEREPVVSFDDVN    120
tr|L7UXZ2|L7UXZ2_ANACO      ---------------------------------------------------PVVSFDDVN    9
sp|P80884|ANAN_ANACO        FKNNVNHIETFNNRNGNSYTLGINQFTDMTNNEFVAQYTG-LSLPLNIKREPVVSFDDVD    119
tr|S5VRY7|S5VRY7_ANACO      FKNNVNHIETFNNRSGNSYTLGINQFTDMTDNEFVAQYTG-VSLPLNIEREPVVSFDDVD    119
tr|O81085|O81085_ANACO      FKNNVNHIETFNSRNGNSYTLGINQFTDMTNNEFVAQYTG-VSLPLNIEREPVVSFDDVD    119


sp|P14518|BROM2_ANACO       --AVPQSIDWRDYGAVTSVKNQNPCGACWAFAAIATVESIYKIKKGILEPLSEQQVLDCA    58
tr|O23799|O23799_ANACO      ISAVPQSIDWRDYGAVTSVKNQNPCGACWAFAAIATVESIYKIKKGILEPLSEQQVLDCA    180
tr|O81084|O81084_ANACO      ISAVPQSIDWRDYGAVTSVKNQNPCGACWAFAAIATVESIYKIKKGILEPLSEQQVLDCA    180
tr|O23800|O23800_ANACO      ISAVGQSIDWRDYGAVTEVKDQNPCGSCWAFSAIATVEGIYKIVTGYLVSLSEQEVLDCA    179
tr|F1KD58|F1KD58_ANACO      ISAVGQSIDWRDYGAVTEVKDQNPCGSCWAFSAIATVEGIYKIVTGYLVSLSEQEVLDCA    180
tr|Q7DNA3|Q7DNA3_ANACO      ISAVGQSIDWRDYGAVTEVKDQNPCGSCWAFSAIATVEGIYKIVTGYLVSLSEQEVLDCA    140
tr|O24641|O24641_ANACO      ISAVGQSIDWRDYGAVTEVKDQNPCGSCWAFSAIATVEGIYKIVTGYLVSLSEQEVLDCA    180
sp|O23791|BROM1_ANACO       ISAVPQSIDWRDYGAVNEVKNQNPCGSCWSFAAIATVEGIYKIKTGYLVSLSEQEVLDCA    179
tr|O23801|O23801_ANACO      ISAVPQSIDWRDYGAVNEVKNQNPCGSCWAFAAIATVEGIYKIKTGYLVSLSEQEVLDCA    152
tr|F8UN02|F8UN02_ANACO      ISAVPQSIDWRDYGAVNEVKNQNPCGSCWAFAAIATVEGIYKIKTGYLVSLSEQEVLDCA    180
tr|L7UXZ2|L7UXZ2_ANACO      ISAVPQSIDWRDYGAVNEVKNQNPCGSCWAFAAIATVEGIYKIKTGYLVSLSEQEVLDCA    69
sp|P80884|ANAN_ANACO        ISSVPQSIDWRDSGAVTSVKNQGRCGSCWAFASIATVESIYKIKRGNLVSLSEQQVLDCA    179
tr|S5VRY7|S5VRY7_ANACO      ISAVPQSIDWRNCGAVTSVKNQNPCGSCWAFAAIATVESIYKIKRGYLVSLSEQQVLDCA    179
tr|O81085|O81085_ANACO      ISAVPQSIDWRNYGAVTSVKNHIPCGSCWAFAAIATVESIYKIKRGYLISLSEQQVLDCA    179
                          :* ******: ***..**::  **:**:*::*****.****  * *  ****:*****


sp|P14518|BROM2_ANACO       KGYGCKGGWEFRAFEFIISNKGVASGAIYPYKAAK--GTCKTDGVPNSAYITGYARVPRN    116
tr|O23799|O23799_ANACO      KGYGCKGGWEFRAFEFIISNKGVASGAIYPYKAAK--GTCKTNGVPNSAYITGYARVPRN    238
tr|O81084|O81084_ANACO      KGYGCKGGWEFRAFEFIISNKGVASVAIYPYKAAK--GTCKTNGVPNSAYITGYARVPRN    238
tr|O23800|O23800_ANACO      VSNGCDGGFVDNAYDFIISNNGVASEADYPYQAYE--GDCTANSWPNSAYITGYSYVRSN    237
tr|F1KD58|F1KD58_ANACO      VSNGCDGGFVDNAYDFIISNNGVASEADYPYQAYQ--GDCAANSWPNSAYITGYSYVRSN    238
tr|Q7DNA3|Q7DNA3_ANACO      VSNGCDGGFVDNAYDFIISNNGVASEADYPYQAYQ--GDCAANSWPNSAYITGYSYVRSN    198
tr|O24641|O24641_ANACO      VSNGCDGGFVDNAYDFIISNNGVASEADYPYQAYQ--GDCAANSWPNSAYITGYSYVRSN    238
sp|O23791|BROM1_ANACO       VSYGCKGGWVNKAYDFIISNNGVTTEENYPYLAYQ--GTCNANSFPNSAYITGYSYVRRN    237
tr|O23801|O23801_ANACO      VSYGCKGGWVNKAYDFIISNNGVTTEENYPYQAYQ--GTCNANSFPNSAYITGYSYVRRN    210
tr|F8UN02|F8UN02_ANACO      VSYGCKGGWVNKAYDFIISNNGVTTEENYPYQAYQ--GTCNANSFPNSAYITGYSYVRRN    238
tr|L7UXZ2|L7UXZ2_ANACO      VSYGCKGGWVNKAYDFIISNNGVTTEENYPYQAYQ--GTCNANSFPNSAYITGYSYVRRN    127
sp|P80884|ANAN_ANACO        VSYGCKGGWINKAYSFIISNKGVASAAIYPYKAAK--GTCKTNGVPNSAYITRYTYVQRN    237
tr|S5VRY7|S5VRY7_ANACO      VSYGCDGGWVNKAYDFIISNKGVASAAIYPYKASQ--GTCRTNGVPNSAYITGYTRVQSN    237
tr|O81085|O81085_ANACO      VSYGCDGGWVNKAYDFIISNKGVASAAIYPYKASQGQGTCRINGVPNSAYITGYTRVQSN    239
                          . **.**:  .*:.*****:**::   *** * :  * *  :. ******* *: *  *


sp|P14518|BROM2_ANACO       NESSMMYAVSKQPITVAVDANA-NFQYYKSGVFNGPCGTSLNHAVTAIGYGQDSI-----    170
tr|O23799|O23799_ANACO      NESSMMYAVSKQPITVAVDANA-NFQYYKSGVFNGPCGTSLNHAVTAIGYGQDSNGKKYW    297
```

```
tr|O81084|O81084_ANACO     NESSMMYAVSKQPITVAVDANA-NSQYYNSGVFNGPCGTSLNHAVTAIGYGQDSNGKKYW        297
tr|O23800|O23800_ANACO     DESSMKYAVWNQPIAAAIDASGDNFQYYNGGVFSGPCGTSLNHAITIIGYGQDSSGTQYW        297
tr|F1KD58|F1KD58_ANACO     DESSMKYAVWNQPIAAAIDASGDNFQYYNGGVFSGPCGTSLNHAITIIGYGQD-------        291
tr|Q7DNA3|Q7DNA3_ANACO     DESSMKYAVWNQPIAAAIDASGDNFQYYNGGVFSGPCGTSLNHAITIIGYGQDSSGTQYW        258
tr|O24641|O24641_ANACO     DESSMKYAVWNQPIAAAIDASGDNFQYYNGGVFSGPCGTSLNHAITIIGYGQDSSGTQYW        298
sp|O23791|BROM1_ANACO      DERSMMYAVSNQPIAALIDASE-NFQYYNGGVFSGPCGTSLNHAITIIGYGQDSSGTKYW        296
tr|O23801|O23801_ANACO     DERSMMYAVSNQPIAALIDASE-NFQYYNGGVFSGPCGTSLNHAITIIGYGQDSSGTKYW        269
tr|F8UN02|F8UN02_ANACO     DERSMMYAVSNQPIAALIDASE-NFQYYNGGVFSGPCGTSLNHAITIIGYGQDSSGTKYW        297
tr|L7UXZ2|L7UXZ2_ANACO     DERSMMYAVSNQPIAALIDASE-NFQYYNGGVFSGPCGTSLNHAITIIGYGQDSSGTKYW        186
sp|P80884|ANAN_ANACO       NERNMMYAVSNQPIAAALDASG-NFQHYKRGVFTGPCGTRLNHAIVIIGYGQDSSGKKFW        296
tr|S5VRY7|S5VRY7_ANACO     NERSMMYAVSNQPIAASIEASG-DFQHYKRGVFSGPCGTSLNHAITIIGYGQDSSGKKFW        296
tr|O81085|O81085_ANACO     NERSMMYAVSNQPIAASIEASG-DFQHYKRGVFSGPCGTSLNHAITIIGYGQDSSGKKFW        298
                         :* .* *** :***:. ::*.  : *:*: ***.***** ****:. ******

sp|P14518|BROM2_ANACO      IYPKKWGAKWGEAGYIRMARDVSSSSGICGIAIDPLYPTLEE-----------------212
tr|O23799|O23799_ANACO     IVKNSWGARWGEAGYIRMARDVSSSSGICGIAIDSLYPTLESRANVEAIKMVSESRSSV        356
tr|O81084|O81084_ANACO     IVKNSWGARWGEAGYIRMARDVSSSSGICGIAIDSLYPTLESRANVEAIKMVSESRSSV        356
tr|O23800|O23800_ANACO     IVKNSWGSSWGERGYVRMARGVS-SSGLCGIAMDPLYPTLQSGA---------------        340
tr|F1KD58|F1KD58_ANACO     -----------------------------------------------------------        291
tr|Q7DNA3|Q7DNA3_ANACO     IVKNSWGSSWGERGYIRMARGVS-SSGLCGIAMDPLYPTLQSGANVAVIKMVSKT----        312
tr|O24641|O24641_ANACO     IVKNSWGSSWGERGYIRMARGVS-SSGLCGIAMDPLYPTLQSGANVAVIKMVSKT----        352
sp|O23791|BROM1_ANACO      IVRNSWGSSWGEGGYVRMARGVSSSSGVCGIAMAPLFPTLQSGANAEVIKMVSET----351
tr|O23801|O23801_ANACO     IVRNSWGSSWGEGGYVRMARGVSSSSGACGIAMSPLFPTLQSGANVEVIKMVSET----        324
tr|F8UN02|F8UN02_ANACO     IVRNSWGSSWGEGGYVRMARGVSSSSGACGIAMSPLFPTLQSGANVEVIKMVSET----        352
tr|L7UXZ2|L7UXZ2_ANACO     IVGNSWGSSWGEGGYVRMARGVSSSSGACGIAMSPLFPTLQSGANVEVIKMVSET----        241
sp|P80884|ANAN_ANACO       IVRNSWGAGWGEGGYIRLARDVSSSFGLCGIAMDPLYPTLQSGPSVEVI----------345
tr|S5VRY7|S5VRY7_ANACO     IVRNSWGASWGERGYIRMARDVSSSSGLCGIAIRPLYPTLQSGANVEVIKMVSESQSSV        355
tr|O81085|O81085_ANACO     IVRNSWGASWGERGYIRMARDVSSSSGLCGIAIRPLYPTLQSGANVEVIKMVSESRSSV        357
```

Figure S1. Multiple sequence alignment of the 14 sequences reported in the Allergome database as Ana c 2. The sequences used for this alignment are those provided by UniProt, which include the signal and propart regions. The sequence regions removed in the mature proteins are highlighted with a grey background. A glycosylation site (N-X-S) is observed in the first three aligned sequences and it is highlighted in dark green. The three residues involved in the catalytic activity have been highlighted in yellow. The asterisks, colons, and dots indicate the identical amino acid residues, conserved substitutions, and semiconserved substitutions, respectively.

Table S1. Amino acid sequence identity (%) between different isoforms of Ana c 2. The accession number of each isoform is reported. The identity values were calculated with the Clustal O algorithm on the UniProt website, using the amino acid sequences aligned in Figure S1.

	P14518	O23799	O81084	O23800	F1KD58	Q7DNA3	O24641	O23791	O23801	F8UN02	L7UXZ2	P80884	S5VRY7	O81085
**P14518**	100													
**O23799**	95.75	100												
**O81084**	94.34	98.03	100											
**O23800**	68.72	76.99	77.29	100										
**F1KD58**	69.05	78.97	79.31	97.24	100									
**Q7DNA3**	69.19	76.21	76.21	97.00	100	100								
**O24641**	69.19	78.06	78.35	97.35	100	100	100							
**O23791**	69.81	77.49	77.49	87.02	87.20	85.48	86.57	100						
**O23801**	70.28	77.16	77.16	87.18	87.40	86.77	87.00	97.53	100					
**F8UN02**	70.28	79.26	79.55	88.20	90.00	87.78	89.17	96.30	98.15	100				
**L7UXZ2**	70.28	73.44	73.44	86.90	87.15	86.67	86.67	97.51	99.59	99.59	100			
**P80884**	75.94	81.16	80.87	76.65	79.93	77.63	79.36	79.71	79.87	81.16	76.60	100		
**S5VRY7**	76.89	83.10	82.82	84.07	84.78	82.26	84.29	84.33	84.26	85.47	81.33	88.41	100	
**O81085**	75.94	83.66	83.38	83.78	84.43	81.94	84.00	84.33	84.26	85.47	80.50	87.83	97.46	100

Table S2. Immunological and clinical data of the 133 patients with specific IgE towards the seven papain-like cysteine proteases analyzed in this study. In addition, the Table shows some details of two patients reporting allergy symptoms after exposure to fig, but they were IgE negative to Fic c Ficin. Patients were selected from a random population of 341 individuals reporting allergy symptoms and subjected to IgE detection with the FABER test.

**Patient details**			**FABER 244 (FIU)***							**Allergy reaction**				
	Sex	Years	Act d 1	Ana c 2	Cari p 2	Cari p Pap [a]	Der p 1	Der f 1	Fic c Fic [b]	Kiwi	Ananas	Papaya	HDM [c]	Fig
1	M	15.8	1.10	0.67	0	0	0	0	0	0	0	0	0	0
2	M	6.3	0.66	0.44	0	0	0	0	0	0	0	0	0	0
3	F	10.2	0.92	2.88	0	0.62	0	0	0	1	0	0	0	0
4	F	28.7	0	24.05	26.32	0	0	0	0	0	0	0	0	0
5	M	22.2	0	1.47	13.94	3.18	0	0	0	0	0	0	0	0
6	M	6.6	0	0.65	0.43	19.15	9.64	11.13	0	0	0	0	0	0
7	M	48.5	0	5.24	2.48	0	1.24	0	0	0	0	0	1	0
8	M	3.6	0	1.38	0.92	0	3.21	8.50	0	0	0	0	1	0
9	M	30.0	0	0	6.25	0	0	0	0	0	0	0	0	0
10	M	49.7	0	0	7.30	0	0	0	0	0	0	0	0	0
11	M	37.8	0	0	0.75	0	0	0	0	0	0	0	0	0
12	F	33.1	0	0	2.24	0	0	0	0	0	0	0	0	0
13	F	37.7	0	1.20	0	0	0.72	0	0	0	0	0	1	0
14	F	55.1	0	0	0	3.52	1.54	0.91	0	0	0	0	1	0
15	F	67.8	0	0	0	0.72	0	0	2.14	0	0	0	0	0
16	F	41.5	0	1.52	0	0	0	0	3.82	0	0	0	0	0

17	F	20.1	0	4.60	0	0	0	0	4.84	0	0	0	0	0
18	F	10.0	0	0	0	0	0	0	2.87	0	0	0	0	0
19	F	52.1	0	0	0	0	0	0	4.24	0	0	0	0	0
20	F	51.6	0	0	0	0	0	0	2.29	0	0	0	0	0
21	F	25.4	0	0	0	0	0	0	5.16	0	0	0	0	0
22	F	34.0	0	0	0	0	0	0	4.96	0	0	0	0	0
23	F	16.7	0	0	0	0	0	0	4.03	0	0	0	0	0
24	F	26.3	0	0	0	0	0	0	3.98	0	0	0	0	0
25	F	18.8	0	0	0	0	0	0	2.15	0	0	0	0	0
26	F	55.8	0	0	0	0	0	0	3.04	0	0	0	0	0
27	F	38.6	0	0	0	0	0	0	3.52	0	0	0	0	0
28	F	34.8	0	0	0	0	0	0	2.95	0	0	0	0	0
29	F	44.7	0	0	0	0	0	0	3.01	0	0	0	0	0
30	M	76.9	0	0	0	0	0.67	0	2.34	0	0	0	0	0
31	M	10.7	0	0	0	0	17.10	14.35	3.01	0	0	0	1	0
32	F	25.9	0	0	0	0	2.10	10.35	3.13	0	0	0	1	0
33	M	12.9	0	0	0	0	3.46	12.23	2.84	0	0	0	1	0
34	F	10.7	0	0	0	0	37.75	67.87	3.37	0	0	0	1	0
35	F	2.1	0	0	0	0	7.43	7.44	3.74	0	0	0	1	0
36	F	30.3	0	0	0	0	1.69	0.91	0	0	0	0	1	0
37	M	30.6	0	0	0	0	2.32	5.87	0	0	0	0	1	0
38	M	24.9	0	0	0	0	0	3.33	4.42	0	0	0	1	0
39	M	38.0	0	0.91	0	0	0	0	3.98	0	0	0	0	0
40	F	36.5	0	2.43	0	0	0	0	4.85	0	0	0	0	0
41	M	45.9	0	0	0	0	0	0	25.58	0	0	0	0	0
42	F	50.1	0	0	0	0	0	0	4.13	0	0	0	0	0
43	M	70.2	0	0	0	0	0	0	3.73	0	0	0	0	0
44	M	59.1	0	0	0	0	0	0	3.19	0	0	0	0	0
45	M	11.3	0	0	0	0	0	0	3.60	0	0	0	0	0
46	F	44.6	0	0	0	0	3.21	0	0	0	0	0	1	0
47	F	53.9	0	0	0	0	1.69	0	0	0	0	0	1	0
48	F	36.5	0	0	0	0	1.39	0	0	0	0	0	1	0
49	M	60.5	0	0	0	0	0.90	0	0	0	0	0	1	0
50	M	9.1	0	0	0	0	0.94	0	0	0	0	0	1	0
51	M	64.5	0	0	0	0	5.29	0	0	0	0	0	1	0
52	M	23.5	0	0	0	0	2.08	2.69	0	0	0	0	1	0
53	M	30.6	0	0	0	0	2.32	5.19	0	0	0	0	1	0
54	M	27.7	0	0	0	0	3.20	19.57	0	0	0	0	1	0
55	M	25.8	0	0	0	0	5.25	5.25	0	0	0	0	1	0
56	M	32.5	0	0	0	0	1.67	1.02	0	0	0	0	1	0
57	M	11.8	0	0	0	0	1.02	1.02	0	0	0	0	1	0
58	M	21.0	0	0	0	0	1.81	1.15	0	0	0	0	1	0

59	M	10.0	0	0	0	0	2.18	1.86	0	0	0	0	1	0
60	M	20.5	0	0	0	0	0.91	1.18	0	0	0	0	1	0
61	M	25.8	0	0	0	0	5.25	5.25	0	0	0	0	1	0
62	M	10.0	0	0	0	0	0.62	1.55	0	0	0	0	1	0
63	M	12.8	0	0	0	0	7.58	13.65	0	0	0	0	1	0
64	M	15.8	0	0	0	0	9.02	19.70	0	0	0	0	1	0
65	M	30.8	0	0	0	0	1.80	2.25	0	0	0	0	1	0
66	M	3.3	0	0	0	0	2.33	6.53	0	0	0	0	1	0
67	M	45.3	0	0	0	0	4.95	1.32	0	1	0	0	1	0
68	F	13.7	0	0	0	0	3.01	4.04	0	0	0	0	1	0
69	F	11.7	0	0	0	0	26.08	22.70	0	0	0	0	0	0
70	F	43.6	0	0	0	0	2.35	4.38	0	0	0	0	1	0
71	F	15.6	0	0	0	0	4.91	9.48	0	0	0	0	1	0
72	M	39.1	0	3.83	0	0	2.26	0.68	0	0	0	0	1	0
73	F	5.2	0	1.17	0	0	0.71	15.82	0	0	0	0	1	0
74	F	15.7	0	7.62	0	0	0.32	0.44	0	0	0	0	1	0
75	F	38.4	0	2.70	0	0	0.73	0.91	0	0	0	0	1	0
76	M	13.5	0	0.67	0	0	5.81	8.21	0	0	0	0	1	0
77	M	29.6	0	5.32	0	0	0.55	1.04	0	0	0	0	1	1
78	F	11.6	0	0	0	0	4.88	7.56	0	0	0	0	1	0
79	F	18.1	0	0	0	0	1.27	0.64	0	0	0	0	1	0
80	M	11.0	0	0	0	0	0.91	1.32	0	0	0	0	1	0
81	M	12.4	0	0	0	0	0.46	0.52	0	0	0	0	1	0
82	M	16.1	0	0	0	0	2.93	2.92	0	0	0	0	1	0
83	F	32.6	0	0	0	0	1.32	3.49	0	0	0	0	1	0
84	F	13.1	0	0	0	0	0.22	0	0	0	0	0	1	0
85	F	39.1	0	0	0	0	0.57	1.97	0	0	0	0	1	0
86	F	39.2	0	0	0	0	0.20	1.05	0	0	0	0	1	0
87	F	47.1	0	0	0	0	0.91	1.17	0	0	0	0	1	0
88	F	14.5	0	0	0	0	0.55	0.98	0	0	0	0	1	0
89	F	45.1	0	0	0	0	2.48	1.16	0	0	0	0	1	0
90	F	48.7	0	0	0	0	0.49	0.55	0	0	0	0	1	0
91	F	33.9	0	0	0	0	0.91	1.21	0	0	0	0	1	0
92	F	34.6	0	0	0	0	2.64	2.55	0	0	0	0	1	0
93	F	8.1	0	0	0	0	0.72	5.39	0	0	0	0	1	0
94	F	16.5	0	0	0	0	2.05	0.57	0	0	0	0	1	0
95	F	47.4	0	0	0	0	0.65	1.84	0	0	0	0	1	0
96	F	9.3	0	0	0	0	0.70	2.01	0	0	0	0	1	0
97	F	36.7	0	0	0	0	0.37	0.48	0	0	0	0	1	0
98	F	45.6	0	0	0	0	0.46	0.60	0	0	0	0	1	0
99	F	20.4	0	0	0	0	0.46	0.37	0	0	0	0	1	0
100	F	42.1	0	0	0	0	0	0.49	0	0	0	0	1	0

101	F	38.0	0	0	0	0	0	1.48	0	0	0	0	1	0
102	F	39.5	0	0	0	0	0	2.25	0	0	0	0	1	0
103	F	51.0	0	0	0	0	0	0.61	0	0	0	0	1	0
104	F	25.5	0	0	0	0	0	0.38	0	0	0	0	1	0
105	F	23.5	0	0	0	0	0	0.82	0	0	0	0	1	0
106	F	74.6	0	0	0	0	0	0.79	0	0	0	0	1	0
107	F	49.6	0	0	0	0	0	0.72	0	0	0	0	1	0
108	M	20.5	0	0	0	0	0	0.48	0	0	0	0	1	0
109	M	12.1	0	0	0	0	0	0.48	0	0	0	0	1	0
110	M	24.2	0	0	0	0	0	0.41	0	0	0	0	1	0
111	M	37.6	0	0	0	0	0	2.04	0	0	0	0	1	0
112	M	70.9	0	0	0	0	0	0.92	0	0	0	0	1	0
113	M	12.6	0	1.52	0	0	0	0.51	0	0	0	0	1	0
114	M	48.0	0	1.25	0	0	0	0.83	0	0	0	0	1	0
115	M	29.5	0	10.31	0	0	0	0	0	0	0	0	0	0
116	M	15.9	0	9.32	0	0	0	0	0	0	0	0	0	0
117	M	25.2	0	1.06	0	0	0	0	0	0	0	0	0	0
118	M	40.2	0	2.32	0	0	0	0	0	0	0	0	0	0
119	M	83.3	0	45.10	0	0	0	0	0	0	0	0	0	0
120	M	22.7	0	1.07	0	0	0	0	0	0	0	0	0	0
121	M	21.6	0	1.07	0	0	0	0	0	0	0	0	0	0
122	F	27.7	0	4.35	0	0	0	0	0	0	0	0	0	0
123	F	35.8	0	0.66	0	0	0	0	0	0	0	0	0	0
124	F	35.0	0	2.91	0	0	0	0	0	0	0	0	0	0
125	F	16.9	0	0.97	0	0	0	0	0	0	0	0	0	0
126	F	52.0	0	0.79	0	0	0	0	0	0	0	0	0	0
127	F	25.0	0	0.34	0	0	0	0	0	0	0	0	0	0
128	F	17.0	0	30.57	0	0	0	0	0	0	0	0	0	0
129	F	26.0	0	5.08	0	0	0	0	0	0	0	0	0	0
130	F	36.5	0	18.91	0	0	0	0	0	0	0	0	0	0
131	F	29.2	0	0.84	0	0	0	0	0	0	0	0	0	0
132	M	24.4	0	2.19	0	0	0	0	0	0	0	0	0	0
133	M	8.1	0	0.77	0	0	0	0	0	0	0	0	0	0
153	M	59.8	0	0	0	0	0	0	0	0	0	0	0	1
252	F	64.9	0	0	0	0	0	0	0	0	0	0	0	1

[a] Cari p Papain; [b] Fic c Ficin [c]allergy symptoms towards House Dust Mite (HDM) on the basis of the clinical history. * FIU, FABER International Units, positive value FIU≥0.01.
